# Comparative study on the predictive value of TyG, TyG-BMI, and TG/HDL-C for progression-free survival in patients with locally advanced nasopharyngeal carcinoma

**DOI:** 10.3389/fnut.2025.1657646

**Published:** 2025-09-12

**Authors:** Zhehao Xiao, Zhuowei Liang, Weiling Chen, Hejing Huang, Song Qu

**Affiliations:** ^1^Department of Radiation Oncology, Guangxi Medical University Cancer Hospital, Nanning, Guangxi, China; ^2^Key Laboratory of Early Prevention and Treatment for Regional High Frequency Tumor (Guangxi Medical University), Ministry of Education, Nanning, Guangxi, China

**Keywords:** nasopharyngeal carcinoma, locally advanced, TyG, TyG-BMI, TG/HDL-C

## Abstract

**Background:**

This study aimed to explore the relationship between the triglyceride glucose (TyG) index, triglyceride glucose-body mass index (TyG-BMI), and triglyceride-to-high-density lipoprotein cholesterol ratio (TG/HDL-C) of locally advanced nasopharyngeal carcinoma (LA-NPC) and progression-free survival (PFS) to investigate their potential as prognostic indicators.

**Methods:**

This research involved a retrospective analysis of data pertaining to patients with LA-NPC from the Guangxi Medical University Cancer Hospital. The analysis categorized patients into a progression group and a control group according to their disease control status. The correlation of three indicators with PFS was established utilizing the Cox proportional hazards model, Kaplan–Meier (K-M) analysis, and restricted cubic spline (RCS) analysis. Three predictive models were developed based on the three indicators, and their predictive ability was assessed.

**Results:**

TyG, TyG-BMI, and TG/HDL-C are independent predictors of PFS in LA-NPC patients, and all exhibit a non-linear relationship. Patients in the high TyG, TyG-BMI, and TG/HDL-C groups have significantly lower PFS compared to those in the low groups, and this effect persists after adjusting for confounding factors. A multivariate analysis confirmed that lactate dehydrogenase (LDH) and EBV_DNA are also independent prognostic factors for PFS. The models that utilize these indicators outperform traditional tumor node metastasis (TNM) staging, with the TyG-based model demonstrating the strongest predictive ability for PFS.

**Conclusion:**

TyG, TyG-BMI, and TG/HDL-C are potential prognostic biomarkers for the evaluation of PFS in individuals diagnosed with LA-NPC. Our research underscores the potential of these three indices to be utilized to enhance prognostic assessment and customize treatment strategies in the management of LA-NPC.

## Introduction

Nasopharyngeal carcinoma (NPC) is a neoplastic malignancy located in the head-and-neck region, with a prevalence that is particularly notable in Southeast Asia, ranking 23rd in global cancer incidence (1). Similar to many other cancers, the early symptoms of NPC are often subtle, and the vast majority of patients are diagnosed at a locally advanced stage (2). Despite continuous improvements in radiotherapy techniques, approximately 30–40% of individuals diagnosed with locally advanced NPC (LA-NPC) experience local recurrence or metastasis after receiving radical treatment, and the survival prognosis for these patients is not satisfactory (3). The TNM system can reflect the anatomical extent of the patient’s lesions, but it does not capture all of the information about the tumor microenvironment in NPC. The recently proposed ecological theory of NPC suggests that it is essential to thoroughly evaluate a range of patient-specific factors that could influence NPC, encompassing their lipid metabolic profiles and nutritional status, to achieve a comprehensive and personalized prognostic assessment for patients with LA-NPC (4).

Insulin resistance (IR) is acknowledged as a significant risk factor for various metabolic disorders and cardiovascular diseases (5). Epidemiological research has demonstrated that factors linked to IR are associated with an elevated risk of cancer and a negative prognosis for cancer outcomes (6). Preclinical research evidence has found that some drugs that reduce IR also possess antitumor activity, which has the potential to impede the invasion and migration of NPC cells, thereby suppressing the progression of NPC (7). The hyperinsulinemic-euglycemic clamp technique is considered the definitive standard for the diagnosis of IR. However, its lengthy duration, the need for frequent blood draws, and high cost limit its application in clinical and epidemiological studies. In contrast, the fasting triglyceride glucose (TyG) index is inexpensive and easily accessible, and it has been widely used in clinical practice. It can serve as a comprehensive indicator of the levels of carbohydrate metabolism and lipid metabolism (8). Additionally, the combined markers of TyG and body mass index (BMI), namely the TyG-BMI index and the triglyceride-to-high-density lipoprotein cholesterol (TG/HDL-C) ratio, are also simple, effective, and clinically useful alternative markers for identifying the metabolic level and nutritional status (9, 10). Some studies have indicated that these indicators have predictive value for the occurrence and prognosis of malignant tumors (11, 12). To date, there exists a lack of scholarly investigation regarding the relationship between TyG, TyG-BMI, TG/HDL-C, and the prognosis of NPC. Therefore, this research aimed to investigate the influence of pre-treatment metabolic indicators on the prognosis of patients diagnosed with LA-NPC, providing insights for clinical prognosis prediction in such patients.

## Materials and methods

### Patient screening

This investigation is retrospective in nature and involves the collection of data from a cohort of 761 patients diagnosed with LA-NPC at Guangxi Medical University Cancer Hospital between January 2015 and June 2021. The inclusion criteria for patients were as follows: (1) clear diagnosis of NPC through histopathological examination; (2) staging according to the eighth edition of the American Joint Committee on Cancer (AJCC) as T3-4N0-1M0 or T1-4N2-3M0; (3) Eastern Cooperative Oncology Group (ECOG) score of 0–1; and (4) age between 18 and 70 years. The exclusion criteria for patients were as follows: (1) presence of other cancers; (2) incomplete follow-up information; and (3) missing key pre-treatment laboratory indicators such as triglyceride (TG) and fasting blood glucose (FBG).

### Data collection

We collected pre-treatment laboratory test results for all patients, including WBC, hemoglobin (HB), platelet (PLT), neutrophilic granulocyte (NE), total cholesterol (TC), TG, HDL, low-density lipoprotein (LDL), FBG, and EBV-DNA copy number. Population variable characteristics included patients’ height, weight, history of smoking and alcohol, history of cardiovascular diseases, diabetes, and hypertriglyceridemia. Treatment-related data included the methods received by patients and the specific details of each treatment plan administered. Additionally, we obtained each patient’s disease control and survival status from the case follow-up system. Progression-free survival (PFS) was defined as the duration from the point of diagnosis to the initial indication of disease progression or mortality. The cutoff date for follow-up in this study was 30 June 2024. Medical records of all cases were anonymized and de-identified before analysis. This study has been approved by the Ethics Committee of Guangxi Medical University Cancer Hospital (protocol code KY2024883) and strictly complied with the Helsinki Declaration. The Ethics Committee of Guangxi Medical University Cancer Hospital agreed to exempt patients from signing informed consent.

### Definition and calculation of indicators


$$\begin{array}{l} \mathrm{TYG}\;\text{index}=\ln[(\text{fasting}\;\mathrm{TG})\times \mathrm{FBG}/2]\;(\text{mmol}/L);\mathrm{BMI}\\ =\text{weight}\;(\mathrm{KG})÷{\text{height}}^{2}\;(M);\mathrm{TYG}-\mathrm{BMI}\;\text{index}={\mathrm{TYG}}^{*}\;\mathrm{BMI}. \end{array}$$

### Statistical analysis

Continuous variables were analyzed using the Mann–Whitney U-test, and the results were recorded as the median within the interquartile range. Categorical variables were analyzed using the chi-square test or Fisher’s exact test and recorded as percentages (%). The relationship between the TyG index, TyG-BMI, and TG/HDL-C ratio with PFS was determined using the Kaplan–Meier (K-M) curves and the Cox proportional hazards models. Additionally, the variance inflation factor (VIF) was used to test for multicollinearity, with a VIF of <5 indicating no multicollinearity among variables, ensuring the independence of variables in the study. Restricted cubic spline (RCS) was used to further explore the dose–response relationship between the three indices and PFS. Finally, we constructed three risk prediction models based on the aforementioned three indices, validating the accuracy of the models using calibration curves; the receiver operating characteristic (ROC) curve analysis was performed to compare the predictive ability, sensitivity, and specificity of the three models for patient PFS. Decision curve analysis (DCA) was used to evaluate the clinical net benefit of the models. A *p*-value of < 0.05 was considered statistically significant. All statistical analyses were performed using SPSS (version 25.0) and R (version 4.3.1) software.

## Results

### Patients

**Figure 1:**
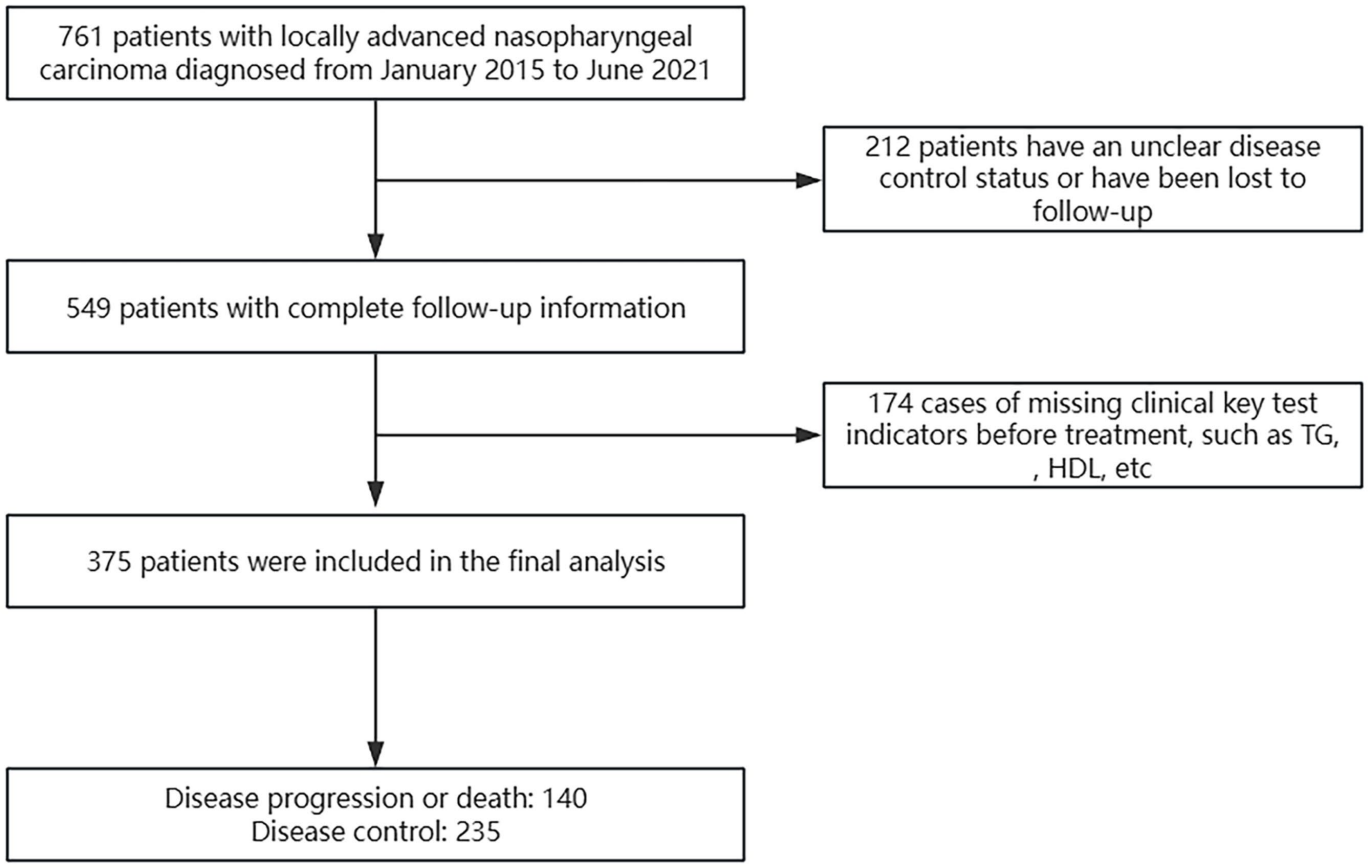
Flowchart of patient inclusion.

A total of 761 patients were evaluated using the electronic medical record system, excluding 212 patients without complete follow-up information and 174 patients with missing pre-treatment laboratory test results. Ultimately, we included 375 patients with LA-NPC (Figure 1).

Among the 375 patients, 271 (72.3%) were men, with a median age of 46 years and a median follow-up duration of 59.3 months. As of 30 June 2024, 140 patients experienced disease progression or death, while the remaining 235 patients had stable disease. All patients received chemotherapy and radiotherapy, with only two patients undergoing induction chemotherapy (IC) combined with radiotherapy, while the remaining patients received IC combined with concurrent chemoradiotherapy (CCRT). All radiotherapy methods used intensity-modulated radiation therapy (IMRT), with a radiation dose of gross tumor volume (GTV): 68–74 Gy/30–34 fractions. Statistically significant variations were observed in the expression levels of LDH, TG, HDL, TYG, TYG-BMI, TG/HDL-C, and EBV-DNA, as well as clinical staging between the two groups of patients, while no statistical differences were found in other variables (Table 1).

**Table 1:** Patient demographics and baseline characteristics.

Characteristics	Disease status			*p*-value
	Overall, *N* = 375	Controlled, *N* = 235	Progressive, *N* = 140	
Sex^1^				0.780^3^
Female	104 (27.7%)	64 (27.2%)	40 (28.6%)	
Male	271 (72.3%)	171 (72.8%)	100 (71.4%)	
Age^1^	46 (39, 53)	43 (38, 53)	49 (40, 53)	0.052^4^
ECOG^1^				0.238^3^
0	273 (72.8%)	176 (74.9%)	97 (69.3%)	
1	102 (27.2%)	59 (25.1%)	43 (30.7%)	
Smoking^1^				0.227^3^
No	250 (66.7%)	162 (68.9%)	88 (62.9%)	
Yes	125 (33.3%)	73 (31.1%)	52 (37.1%)	
Alcohol^1^				0.767^3^
No	278 (74.1%)	173 (73.6%)	105 (75.0%)	
Yes	97 (25.9%)	62 (26.4%)	35 (25.0%)	
Cardiovascular disease^1^				0.530^2^
No	347 (92.5%)	219 (93.2%)	128 (91.4%)	
Yes	28 (7.5%)	16 (6.8%)	12 (8.6%)	
Diabetes^1^				0.376^5^
No	363 (96.8%)	229 (97.4%)	134 (95.7%)	
Yes	12 (3.2%)	6 (2.6%)	6 (4.3%)	
Hypertriglyceridemia^1^				
No	344 (91.7%)	217 (92.3%)	127 (90.7%)	0.580
Yes	31 (8.3%)	18 (7.7%)	13 (9.3%)	
IC regimen^1^				0.914^3^
GP	146 (38.9%)	91 (38.7%)	55 (39.3%)	
TPF	229 (61.1%)	144 (61.3%)	85 (60.7%)	
CCRT^1^				0.531^5^
No	2 (0.5%)	2 (0.9%)	0 (0.0%)	
Yes	373 (99.5%)	233 (99.1%)	140 (100.0%)	
AC^1^				>0.999^5^
No	363 (96.8%)	227 (96.6%)	136 (97.1%)	
Yes	12 (3.2%)	8 (3.4%)	4 (2.9%)	
Immunotherapy^1^				0.753^5^
No	364 (97.1%)	227 (96.6%)	137 (97.9%)	
Yes	11 (2.9%)	8 (3.4%)	3 (2.1%)	
Targeted therapy^1^				0.1403
No	270 (72.0%)	163 (69.4%)	107 (76.4%)	
Yes	105 (28.0%)	72 (30.6%)	33 (23.6%)	
LDH^2^ (U/L)	180 (158, 222)	175 (152, 201)	201 (168, 260)	**<0.001** ^**4**^
ALB^2^ (g/L)	39.2 (37.1, 41.3)	39.3 (37.1, 41.4)	39.0 (37.1, 41.1)	0.600^4^
TC^2^ (mmol/L)	4.82 (4.32, 5.44)	4.82 (4.35, 5.42)	4.81 (4.27, 5.52)	0.760^4^
TG^2^ (mmol/L)	1.29 (0.99, 1.71)	1.13 (0.85, 1.56)	1.53 (1.22, 1.90)	**<0.001** ^**4**^
HDL^2^ (mmol/L)	1.14 (1.01, 1.32)	1.15 (1.04, 1.33)	1.10 (0.98, 1.31)	**0.010** ^**4**^
LDL^2^ (mmol/L)	3.19 (2.69, 3.72)	3.19 (2.68, 3.65)	3.17 (2.73, 3.78)	0.354^4^
FBG^2^ (mmol/L)	4.61 (4.28, 5.05)	4.59 (4.24, 4.94)	4.68 (4.30, 5.27)	0.079^4^
WBC^2^ (10*9/L)	6.92 (5.80, 8.08)	6.93 (5.77, 8.07)	6.92 (5.84, 8.09)	0.682^4^
HB^2^ (g/L)	137 (127, 147)	139 (127, 148)	136 (127, 144)	0.104^4^
PLT^2^ (10*9/L)	266 (231, 315)	266 (231, 310)	266 (233, 325)	0.378^4^
NE^2^ (10*9/L)	4.17 (3.40, 5.25)	4.16 (3.40, 5.26)	4.19 (3.42, 5.15)	0.705^4^
BMI^2^ (kg/m^2^)	22.99 (21.26, 24.68)	22.86 (21.07, 24.61)	23.05 (21.72, 25.10)	0.149^4^
TyG^2^	6.86 (6.61, 7.20)	6.72 (6.44, 7.09)	7.05 (6.85, 7.32)	**<0.001** ^**4**^
TyG_BMI^2^	157 (142, 176)	150 (139, 171)	164 (151, 180)	**<0.001** ^**4**^
TG/HDL_C^2^	1.10 (0.83, 1.54)	0.98 (0.72, 1.38)	1.35 (1.05, 1.74)	**<0.001** ^**4**^
EBV_DNA^1^ (copy/mL)				**<0.001** ^**3**^
<1,000	250 (66.7%)	179 (76.2%)	71 (50.7%)	
≥1,000	125 (33.3%)	56 (23.8%)	69 (49.3%)	
T_stage^1^				0.580^3^
1	19 (5.1%)	13 (5.5%)	6 (4.3%)	
2	85 (22.7%)	57 (24.3%)	28 (20.0%)	
3	141 (37.6%)	89 (37.9%)	52 (37.1%)	
4	130 (34.7%)	76 (32.3%)	54 (38.6%)	
N_stage^1^				0.169^5^
0	6 (1.6%)	5 (2.1%)	1 (0.7%)	
1	89 (23.7%)	62 (26.4%)	27 (19.3%)	
2	142 (37.9%)	90 (38.3%)	52 (37.1%)	
3	138 (36.8%)	78 (33.2%)	60 (42.9%)	
cTNM^1^				**0.001** ^**3**^
III	124 (33.1%)	92 (39.1%)	32 (22.9%)	
IVA	251 (66.9%)	143 (60.9%)	108 (77.1%)	

^1^n (%); ^2^median (IQR); ^3^Pearson’s chi-square test; ^4^Wilcoxon rank-sum test; ^5^Fisher’s exact test. IC, induction chemotherapy; CCRT, concurrent chemoradiotherapy; AC, adjuvant chemotherapy; LDH, lactate dehydrogenase; ALB, albumin; TC, total Cholesterol; TG, triglyceride; HDL, high-density lipoprotein; LDL, low-density lipoprotein; FBG, fasting blood glucose; WBC, leukocyte; HB, hemoglobin; PLT, platelet; NE, neutrophilic granulocyte; BMI, body mass index; TyG, triglyceride glucose index; TyG-BMI, triglyceride glucose body mass index; TG/HDL-C, triglyceride-to-high-density lipoprotein cholesterol ratio. Bold values indicate statistical significance.

### TyG, TyG-BMI, and TG/HDL-C and their prognosis in LA-NPC

**Figure 2:**
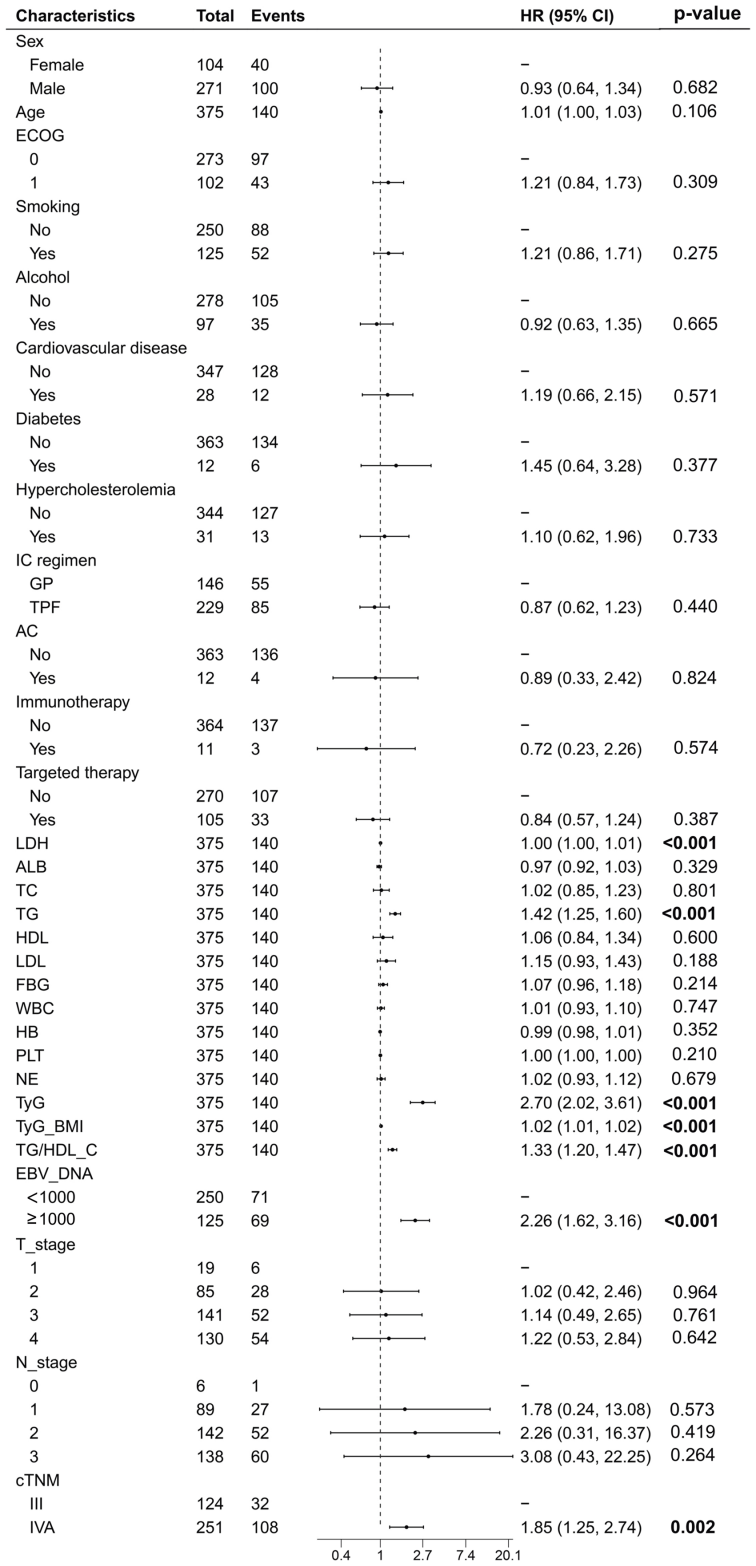
The univariate Cox regression analysis of all variables related to progression-free survival.

The univariate Cox analysis indicated that TyG (HR = 2.70, 95% CI: 2.02–3.61), TyG-BMI (HR = 1.02, 95% CI: 1.01–1.02), and TG/HDL-C (HR = 1.33, 95% CI: 1.20–1.47) were risk factors for patients’ PFS. In addition, pre-treatment LDH (HR = 1.00, 95% CI: 1.00–1.01), EBV-DNA (HR = 2.26, 95% CI: 1.62–3.16), and cTNM staging (HR = 1.85, 95% CI: 1.25–2.74) were also associated with poorer PFS in patients. We performed a collinearity analysis of TyG, TyG-BMI, and TG/HDL-C together with LDH, EBV-DNA, and cTNM staging, and the VIF values were all less than 5, indicating that there was no collinearity between TyG, TyG-BMI, and TG/HDL-C in relation to LDH, EBV-DNA, and cTNM (Figure 2).

**Figure 3:**
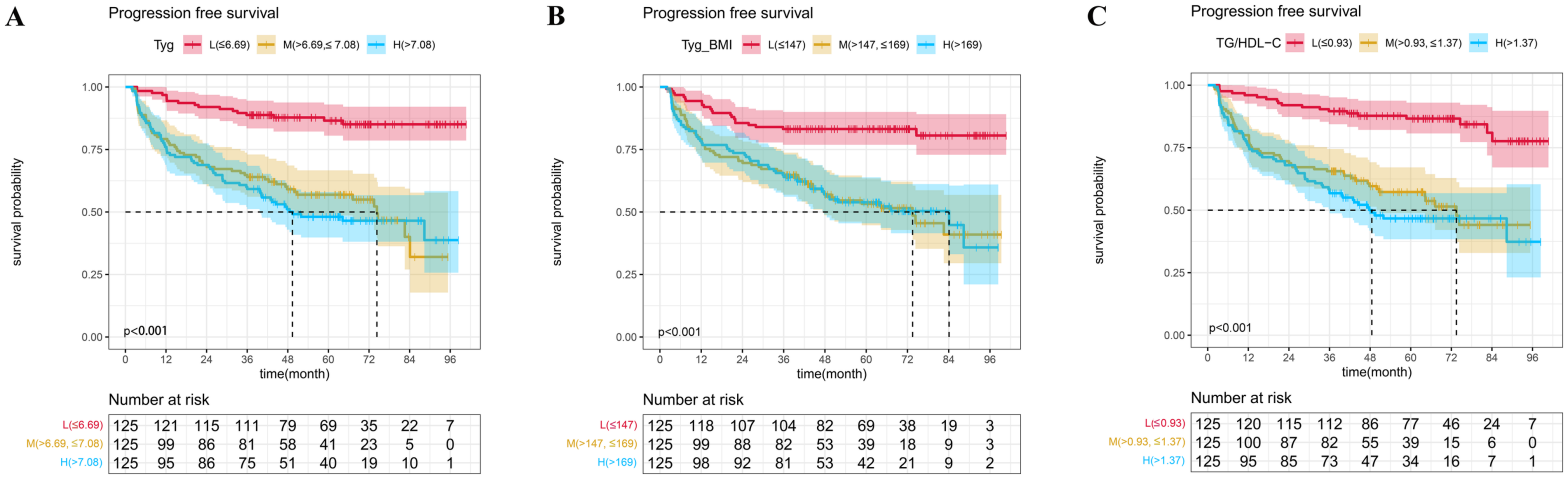
The Kaplan–Meier survival analysis of progression-free survival **(A)** TyG; **(B)** TyG-BMI; and **(C)** TG/HDL-C.

In addition, we established three Cox proportional hazards models for TyG, TyG-BMI, and TG/HDL-C, respectively. The test results of the patients for the aforementioned indicators were arranged in ascending order and divided into three equal parts, categorized as low, medium, and high groups. Model 1 was not adjusted for any variables. Model 2 was a partially adjusted model that accounted for sex, age, ECOG performance status, smoking, alcohol, cardiovascular disease, diabetes, hypertriglyceridemia, chemotherapy regimen, immunotherapy, and targeted therapy. Model 3 was a comprehensively adjusted model that accounted for variables such as sex, age, ECOG performance status, smoking, alcohol, cardiovascular disease, diabetes, hypertriglyceridemia, chemotherapy regimen, immunotherapy, targeted therapy, LDH, ALB, LDL, EBV_DNA, T_stage, and N_stage. The findings indicated that, in the unadjusted model, the partially adjusted model, and the fully adjusted model, TyG, TyG-BMI, and TG/HDL-CI remained prognostic factors for patients’ PFS, whether analyzed as continuous variables or categorical variables (Table 2). The PFS K-M curves of TyG, TyG-BMI, and TG/HDL-C tertiles show that the PFS of patients decreases with an increase in TyG (*p* < 0.001), TyG-BMI (*p* < 0.001), and TG/HDL-C (*p* < 0.001) (Figure 3).

**Table 2:** Association between TyG, TyG_BMI, and TG/HDL_C and PFS (Cox regression).

Characteristics	Model 1			Model 2			Model 3		
	HR^1^	95% CI^1^	*p*-value	HR^1^	95% CI^1^	*p*-value	HR^1^	95% CI^1^	*p*-value
TyG (continuous)	2.70	2.02, 3.61	<0.001	3.69	2.18, 6.26	<0.001	3.42	2.43, 4.81	<0.001
TyG									
L (<6.69)	–	–		–	–		–	–	
M (≥6.69, <7.08)	4.40	2.56, 7.57	<0.001	3.94	2.25, 6.89	<0.001	4.17	2.37, 7.34	<0.001
H (≥7.08)	5.05	2.96, 8.62	<0.001	3.85	2.03, 7.31	<0.001	5.65	3.22, 9.91	<0.001
P for trend			<0.001			<0.001			<0.001
TyG_BMI (continuous)	1.02	1.01, 1.02	<0.001	1.02	1.01, 1.03	<0.001	1.02	1.02, 1.03	<0.001
TyG_BMI									
L (<147)	–	–		–	–		–	–	
M (≥147, <169)	3.34	2.04, 5.46	<0.001	3.40	2.06, 5.61	<0.001	3.70	2.21, 6.20	<0.001
H (≥169)	3.35	2.05, 5.47	<0.001	3.77	2.26, 6.29	<0.001	4.00	2.38, 6.73	<0.001
P for trend			<0.001			<0.001			<0.001
TG/HDL_C (continuous)	1.33	1.20, 1.47	<0.001	1.36	1.21, 1.52	<0.001	1.46	1.29, 1.65	<0.001
TG/HDL_C									
L (<0.93)	–	–		–	–		–	–	
M (≥0.93, <1.37)	4.00	2.37, 6.75	<0.001	4.01	2.36, 6.82	<0.001	4.26	2.47, 7.32	<0.001
H (≥1.37)	4.82	2.88, 8.06	<0.001	4.89	2.88, 8.30	<0.001	5.42	3.15, 9.32	<0.001
P for trend			<0.001			<0.001			<0.001

^1^HR, hazard ratio; CI, confidence interval. Model 1: non-adjusted. Model 2: adjusted for sex, age, ECOG, smoking, alcohol, cardiovascular disease, diabetes, hypertriglyceridemia, chemotherapy regimen, immunotherapy, and targeted therapy. Model 3: adjusted for sex, age, ECOG, smoking, alcohol, cardiovascular disease, diabetes, hypertriglyceridemia, chemotherapy regimen, immunotherapy, targe.ted therapy, LDH, ALB, LDL, EBV_DNA, T_stage, and N_stage.

**Figure 4:**
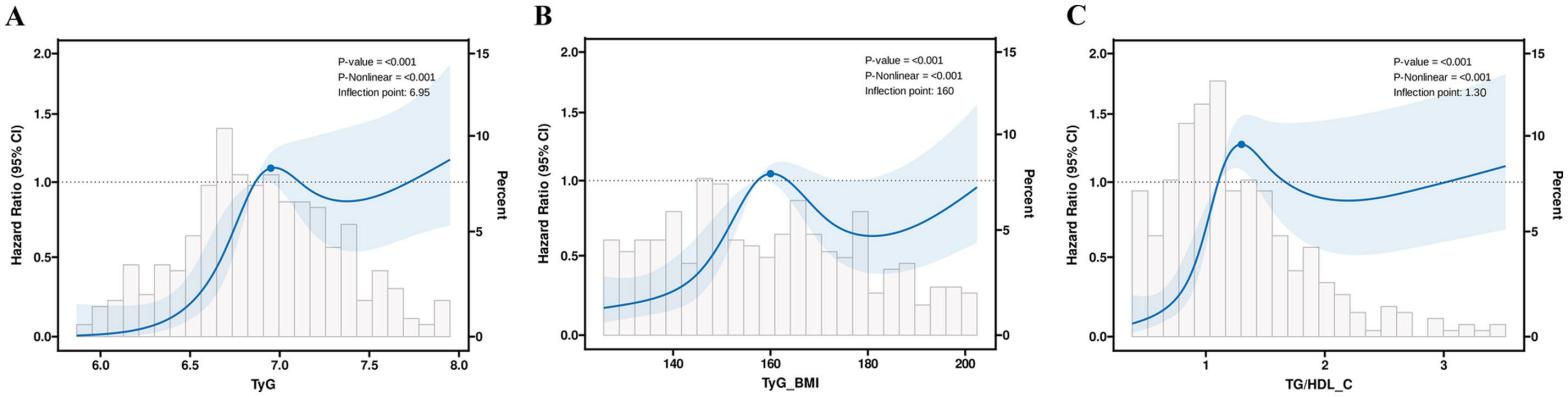
The restricted cubic spline regression analysis of progression-free survival in patients **(A)** TyG; **(B)** TyG-BMI; and **(C)** TG/HDL-C.

A more in-depth examination of the linear correlation between the three aforementioned variables and PFS is warranted. In the complete model, the RCS curve results show that TyG (*p* < 0.001, P-non-linear = 0.001), TyG-BMI (*p* < 0.001, P-non-linear = 0.001), and TG/HDL-C (*p* < 0.001, P-non-linear = 0.001) exhibit a non-linear relationship with patients’ PFS, with the corresponding inflection points being 6.95, 160, and 1.30, respectively (Figure 4).

### Model based on TyG, TyG-BMI, and TG/HDL-C

**Figure 5:**
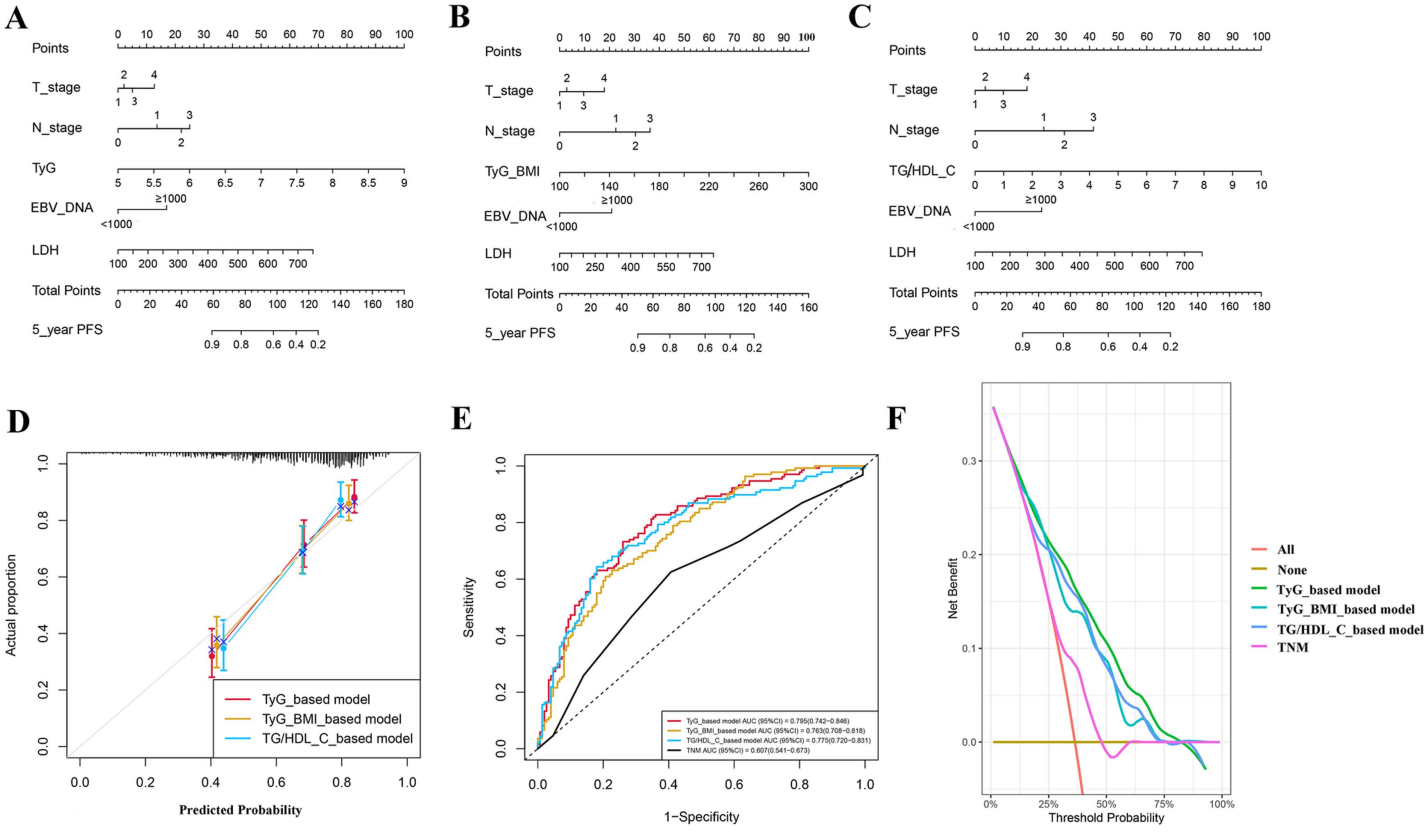
Prediction models for patients’ progression-free survival; **(A)** model based on TyG; **(B)** model based on TyG-BMI; **(C)** model based on TG/HDL-C; **(D)** calibration curve; **(E)** receiver operating characteristic curve; and **(F)** decision curve analysis curve.

We developed three nomograms to predict patient prognosis based on TyG (Figure 5A), TyG-BMI (Figure 5B), and TG/HDL-C (Figure 5C). The nomograms also included pre-treatment LDH, EBV_DNA, T_stage, and N_stage of the patients. The calibration curves indicated that the three nomograms are accurate (Figure 5D). The ROC curves show that the area under the curve (AUC) of the three models is 0.795, 0.763, and 0.775, respectively, all of which are superior to the TNM staging, with the TyG-based model having the highest AUC (Figure 5E). The DCA demonstrate that the clinical net benefit of the TyG-based model surpasses that of the TyG-BMI and TG/HDL-C-based models; however, all three models yield a net benefit exceeding that of the TNM staging system when considered in isolation (Figure 5F).

## Discussion

To the best of our knowledge, this research represents the inaugural examination of the relationship between the TyG, TyG-BMI, and TG/HDL-C ratio with the prognosis of LA-NPC. We found that higher pre-treatment levels of TyG, TyG-BMI, and TG/HDL-C ratio were linked to a reduced PFS in patients, and this relationship remained statistically significant even after controlling for potential confounding variables. Additionally, the TyG index demonstrated a certain advantage in predicting patients’ PFS in relation to the TyG-BMI and TG/HDL-C ratios.

Abnormal lipid metabolism is typically characterized by decreased TC, TG, and HDL-C (13). A substantial body of research evidence indicates that the accumulation of TG is correlated with the occurrence and mortality rates of multiple types of cancer (14, 15). Previous studies have reported that reduced levels of HDL-C are associated with a heightened risk of developing breast cancer in postmenopausal women (16). Furthermore, abnormalities in lipid metabolism are closely related to cancer prognosis. Huang et al. found that elevated TG levels and reduced HDL-C levels are correlated with unfavorable PFS and overall survival (OS) in non-metastatic NPC patients (17). Xie et al. suggested that triglycerides may serve as potential risk factors for the occurrence of eye metastases in male patients diagnosed with NPC (18). Other scholars have also found that elevated levels of HDL-C have been associated with a worse prognosis in patients diagnosed with non-small cell lung cancer (NSCLC) (19). In our research, we observed that TG levels in patients experiencing recurrent metastasis were elevated compared to those in patients with stable disease, and TG is also a prognostic factor affecting the patients’ PFS, which is consistent with previous research findings. Lipids constitute one of the three primary macronutrients. Under conditions of hypoxia and nutrient deficiency, tumor cells typically rely on lipids as a primary source for energy storage, cell membrane construction, and signaling molecules. Consequently, lipid metabolism in neoplastic cells represents a prevalent and significant metabolic characteristic associated with tumor development and progression. The lipid metabolism of tumor cells undergoes abnormal changes during tumor initiation, invasion, and metastasis, including abnormal metabolic changes of lipids such as fatty acids and cholesterol, which may lead to systemic changes in blood lipids. These changes often indicate disease progression and poor prognosis (20). The TG/HDL-C ratio is also a derived indicator based on lipid levels. Prior research has demonstrated that elevated levels of TG/HDL-C serve as an independent prognostic indicator for OS in triple-negative breast cancer (HR: 1.935; 95% CI: 1.032–3.629) (21). A separate retrospective study indicated that patients with NSCLC who exhibited low TG/HDL-C ratios experienced an extended OS (22). This finding is consistent with the results we observed regarding the prognosis of LA-NPC patients. In contrast to a singular indicator, the TG/HDL-C ratio provides a more comprehensive representation of the lipid metabolism status in patients and warrants greater consideration in clinical practice.

Diabetes and elevated FBG have been associated with various cancers (23). The association between elevated FBG levels and cancer prognosis has been a topic of considerable debate. Luo et al. discovered that NSCLC patients exhibiting elevated FBG levels had a 69% higher risk of all-cause mortality compared with those with normal FBG levels (24). Previous research has indicated that women exhibiting elevated FBG levels experience a greater mortality rate from breast cancer compared to those with normal FBG levels (HR = 2.6, 95% CI: 1.2–5.7) (25). Nonetheless, a retrospective investigation carried out by Zhang et al. revealed no substantial association between preoperative FBG levels and OS in patients diagnosed with pancreatic cancer (HR = 1.04, 95% CI: 0.78–1.40) (26). A large cohort study found that no direct correlation exists between diabetes, prediabetes, and the survival outcomes of patients diagnosed with NPC (27). In our research, FBG levels in the LA-NPC group exhibiting progression were marginally elevated compared to those in the non-progression group; however, this observed difference did not achieve statistical significance (*p* = 0.079). Simultaneously, we did not find an association between elevated FBG and patient prognosis. However, it is undeniable that the promoting effect of elevated blood glucose on cancer has been confirmed by many studies. Increased FBG can promote the epithelial–mesenchymal transition (EMT) process, thereby facilitating tumor invasion and metastasis. This facilitative effect can be accomplished through a range of mechanisms, which include the upregulation of N-cadherin expression and the enhancement of transcription levels of Snail and ZEB1 (28). In addition, hyperglycemia has the potential to enhance cellular proliferation and suppress apoptosis through the activation of the NF-κB signaling pathway, while also accelerating cancer progression through promoting angiogenesis (29). It has the capacity to enhance the expression of vascular endothelial growth factor (VEGF) and promote the activation of the VEGF—VEGF receptor-2 (VEGFR2) pathway to facilitate the generation of blood vessels to meet the needs of continuously proliferating tumor cells (30). FBG levels should be an indispensable factor in tumor prognosis assessment.

TyG, as a comprehensive indicator that combines lipid metabolism and glucose metabolism levels, has been confirmed to have a predictive value for cancer risk and act as a supplementary measure of insulin resistance. A meta-analysis showed that a higher TyG index may elevate the likelihood of developing cancer (total effect size = 1.14, 95% CI: 1.08, 1.20) (31). Research by Song et al. indicated that TyG levels are significantly elevated in the early stages of pancreatic ductal adenocarcinoma (PDAC) (32). Our research first found that high TyG is indicative of poor prognosis in LA-NPC patients. Based on the current state of research, many TyG-related indicators have been derived. TyG-BMI is a novel indicator that combines TyG and BMI, which better reflects the nutritional status and overall metabolic level of patients. A large study involving 4,583 participants indicated that an increase in TyG-BMI is independently correlated with the risk of stroke among middle-aged and elderly populations in China (33). Furthermore, an increase in the TyG-BMI index has demonstrated significant utility in the evaluation of the risk and prognosis of NSCLC (34, 35). An elevation in BMI is correlated with the occurrence of multiple types of cancer and is linked to unfavorable prognostic outcomes (36, 37). We also observed poor prognosis in patients with high TyG-BMI in our study. However, it is essential to acknowledge that, within our predictive model, we found that the model based on TyG-BMI had a lower predictive ability than the model based on TyG alone. Low BMI is often associated with malnutrition and cachexia (38), while patients with an appropriate increase in BMI are better able to tolerate anti-tumor treatment, which may counteract some of the risks associated with high BMI.

The most important clinical significance of this study lies in its potential translational value. Pre-treatment TyG, TyG-BMI, and TG/HDL-C, as easily accessible metabolic indicators, can provide an effective risk stratification tool for patients with LA-NPC. First, in terms of follow-up strategies, for high-risk patients with elevated levels of these indicators, more intensive follow-up plans could be considered, such as shortening the intervals between imaging examinations or more frequent monitoring of EBV_DNA, to enable early detection and intervention for disease progression or recurrence. Second, in terms of treatment strategies, these findings provide a basis for exploring personalized adjuvant therapies in the future. Given the potential biological link between insulin resistance and tumor progression, it is worth investigating the feasibility of adding insulin sensitizers (such as metformin) or adjusting the intensity of adjuvant chemotherapy for high-risk patients on top of standard treatment. Finally, in patient management, these indicators can serve as warning signals for lifestyle interventions. Clinicians can use them to provide enhanced dietary and exercise guidance to patients with metabolic abnormalities, which may not only improve cancer prognosis but also benefit the patients’ long-term overall health.

This study is subject to certain limitations. First, it is a retrospective analysis conducted at a single center. Notably, 212 patients were excluded due to missing follow-up information, which may significantly impact the reliability and generalizability of the study results. This reduction in sample size can decrease statistical power, potentially making it unable to detect true clinical differences. Although it is difficult to completely avoid the issue of missing data in retrospective studies, future research should aim to reduce bias by strengthening follow-up management and employing sensitivity analyses. Second, a potential limitation of this study is that we only analyzed the baseline levels of metabolic biomarkers. Since the concentrations of these molecules may change over time, our approach did not capture their dynamic fluctuations. This may affect the accuracy of our assessment of the associations between these biomarkers and clinical outcomes. Future studies involving longitudinal repeated measurements to monitor changes in these metabolites will help to more comprehensively reveal their clinical significance.

## Conclusion

The research identified a notable correlation between the pre-treatment TyG, TyG-BMI, and TG/HDL-C with the PFS of LA-NPC patients and, based on this correlation, three models were established, with the TyG-based model outperforming the other two. Our investigation underscores the promise of these three indicators in enhancing prognostic evaluations and tailoring treatment strategies for locally advanced LA-NPC. Further research is necessary to validate these findings and to investigate the underlying mechanisms related to the prognosis of NPC.

## Data Availability

The raw data supporting the conclusions of this article will be made available by the authors, without undue reservation.

## Glossary

EMT: Epithelial-mesenchymal transition
K-M: Kaplan–Meier
LA-NPC: Locally advanced NPC
LDH: Lactate dehydrogenase
LDL: Low-density lipoprotein
NE: Neutrophilic granulocyte
NPC: Nasopharyngeal carcinoma
NSCLC: Non-small cell lung cancer
OS: Overall survival
PFS: Progression-free survival
PLT: Platelet
RCS: Restricted cubic spline
ROC: Receiver operating characteristic
TC: Total cholesterol
TG: Triglyceride
TyG: Triglyceride glucose
TyG-BMI: Triglyceride glucose-body mass index
TG/HDL-C: Triglyceride-to-high-density lipoprotein cholesterol ratio
VEGF: Vascular endothelial growth factor
VEGFR2: VEGF-VEGF receptor-2
VIF: Variance inflation factor
WBC: Leukocyte

## References

[1] Bray F Laversanne M Sung H Ferlay J Siegel RL Soerjomataram I. Global cancer statistics 2022: GLOBOCAN estimates of incidence and mortality worldwide for 36 cancers in 185 countries. CA Cancer J Clin. (2024) 74:229–63. doi: 10.3322/caac.21834, PMID: 38572751

[2] Juarez-Vignon Whaley JJ Afkhami M Sampath S Amini A Bell D Villaflor VM. Early stage and locally advanced nasopharyngeal carcinoma treatment from present to future: where are we and where are we going? Curr Treat Options Oncol. (2023) 24:845–66. doi: 10.1007/s11864-023-01083-2, PMID: 37145382 PMC10271909

[3] Hui EP Leung SF Au JS Zee B Tung S Chua D. Lung metastasis alone in nasopharyngeal carcinoma: a relatively favorable prognostic group. A study by the Hong Kong nasopharyngeal carcinoma study group. Cancer. (2004) 101:300–6. doi: 10.1002/cncr.20358, PMID: 15241827

[4] Luo W. Nasopharyngeal carcinoma ecology theory: cancer as multidimensional spatiotemporal "unity of ecology and evolution" pathological ecosystem. Theranostics. (2023) 13:1607–31. doi: 10.7150/thno.82690, PMID: 37056571 PMC10086202

[5] Hill MA Yang Y Zhang L Sun Z Jia G Parrish AR. Insulin resistance, cardiovascular stiffening and cardiovascular disease. Metabolism. (2021) 119:154766. doi: 10.1016/j.metabol.2021.154766, PMID: 33766485

[6] Esposito K Chiodini P Colao A Lenzi A Giugliano D. Metabolic syndrome and risk of cancer: a systematic review and meta-analysis. Diabetes Care. (2012) 35:2402–11. doi: 10.2337/dc12-0336, PMID: 23093685 PMC3476894

[7] Sun XJ Zhang P Li HH Jiang ZW Jiang CC Liu H. Cisplatin combined with metformin inhibits migration and invasion of human nasopharyngeal carcinoma cells by regulating E-cadherin and MMP-9. Asian Pac J Cancer Prev. (2014) 15:4019–23. doi: 10.7314/apjcp.2014.15.9.4019, PMID: 24935589

[8] Min Y Wei X Wei Z Song G Zhao X Lei Y. Prognostic effect of triglyceride glucose-related parameters on all-cause and cardiovascular mortality in the United States adults with metabolic dysfunction-associated steatotic liver disease. Cardiovasc Diabetol. (2024) 23:188. doi: 10.1186/s12933-024-02287-y, PMID: 38824550 PMC11144336

[9] Giannini C Santoro N Caprio S Kim G Lartaud D Shaw M. The triglyceride-to-HDL cholesterol ratio: association with insulin resistance in obese youths of different ethnic backgrounds. Diabetes Care. (2011) 34:1869–74. doi: 10.2337/dc10-2234, PMID: 21730284 PMC3142016

[10] Alagiakrishnan K Halverson T. Role of peripheral and central insulin resistance in neuropsychiatric disorders. J Clin Med. (2024) 13:13. doi: 10.3390/jcm13216607, PMID: 39518747 PMC11547162

[11] Cai C Chen C Lin X Zhang H Shi M Chen X. An analysis of the relationship of triglyceride glucose index with gastric cancer prognosis: a retrospective study. Cancer Med. (2024) 13:e6837. doi: 10.1002/cam4.6837, PMID: 38204361 PMC10905246

[12] Li J Chen J Liu H Yan S Wang Y Xing M. Association of the triglyceride-glucose index with the occurrence and recurrence of colorectal adenomas: a retrospective study from China. BMC Public Health. (2024) 24:579. doi: 10.1186/s12889-024-18076-x, PMID: 38395868 PMC10885480

[13] Bian X Liu R Meng Y Xing D Xu D Lu Z. Lipid metabolism and cancer. J Exp Med. (2021) 218:218. doi: 10.1084/jem.20201606, PMID: 33601415 PMC7754673

[14] Häggström C Jonsson H Bjørge T Nagel G Manjer J Ulmer H. Linear age-course effects on the associations between body mass index, triglycerides, and female breast and male liver cancer risk: an internal replication study of 800, 000 individuals. Int J Cancer. (2020) 146:58–67. doi: 10.1002/ijc.32240, PMID: 30815851

[15] Lin F Zheng R Yu C Su Y Yan X Qu F. Predictive role of serum cholesterol and triglycerides in cervical cancer survival. Int J Gynecol Cancer. (2021) 31:171–6. doi: 10.1136/ijgc-2020-001333, PMID: 33051246

[16] Moorman PG Hulka BS Hiatt RA Krieger N Newman B Vogelman JH. Association between high-density lipoprotein cholesterol and breast cancer varies by menopausal status. Cancer Epidemiol Biomarkers Prev. (1998) 7:483–8. 9641492

[17] Huang R Chen K Jiang Y Li L Zhu X. Development of prognostic nomogram based on lipid metabolic markers and lactate dehydrogenase in non-metastatic nasopharyngeal carcinoma. J Inflamm Res. (2023) 16:3093–107. doi: 10.2147/jir.S416801, PMID: 37520664 PMC10378618

[18] Xie Z Shao Y. The predictive value of serum lipids for eye metastases in male nasopharyngeal carcinoma patients. Biosci Rep. (2020) 40:40. doi: 10.1042/bsr20201082, PMID: 32584390 PMC7317591

[19] Siemianowicz K Gminski J Stajszczyk M Wojakowski W Goss M Machalski M. Serum HDL cholesterol concentration in patients with squamous cell and small cell lung cancer. Int J Mol Med. (2000) 6:307–11. doi: 10.3892/ijmm.6.3.307, PMID: 10934294

[20] Jerby L Wolf L Denkert C Stein GY Hilvo M Oresic M. Metabolic associations of reduced proliferation and oxidative stress in advanced breast cancer. Cancer Res. (2012) 72:5712–20. doi: 10.1158/0008-5472.Can-12-2215, PMID: 22986741

[21] Dai D Chen B Wang B Tang H Li X Zhao Z. Pretreatment TG/HDL-C ratio is superior to triacylglycerol level as an independent prognostic factor for the survival of triple negative breast Cancer patients. J Cancer. (2016) 7:1747–54. doi: 10.7150/jca.15776, PMID: 27698913 PMC5039397

[22] Kong L Zhao Q Han Z Xue W Hu Z Niu Z. Prognostic significance of TG/HDL-C and non-HDL-C/HDL-C ratios in patients with non-small cell lung cancer: a retrospective study. J Int Med Res. (2022) 50:3000605221117211. doi: 10.1177/03000605221117211, PMID: 35949158 PMC9373166

[23] Lega IC Lipscombe LL. Review: diabetes, obesity, and Cancer-pathophysiology and clinical implications. Endocr Rev. (2020) 41:41. doi: 10.1210/endrev/bnz014, PMID: 31722374

[24] Luo J Chen YJ Chang LJ. Fasting blood glucose level and prognosis in non-small cell lung cancer (NSCLC) patients. Lung Cancer. (2012) 76:242–7. doi: 10.1016/j.lungcan.2011.10.019, PMID: 22112292

[25] Minicozzi P Berrino F Sebastiani F Falcini F Vattiato R Cioccoloni F. High fasting blood glucose and obesity significantly and independently increase risk of breast cancer death in hormone receptor-positive disease. Eur J Cancer. (2013) 49:3881–8. doi: 10.1016/j.ejca.2013.08.004, PMID: 24011933

[26] Zhang M Hu X Kang Y Xu W Yang X. Association between fasting blood glucose levels at admission and overall survival of patients with pancreatic cancer. BMC Cancer. (2021) 21:131. doi: 10.1186/s12885-021-07859-9, PMID: 33549043 PMC7866692

[27] OuYang PY Su Z Tang J Lan XW Mao YP Deng W. Diabetes, prediabetes and the survival of nasopharyngeal carcinoma: a study of 5,860 patients. PLoS One. (2014) 9:e111073. doi: 10.1371/journal.pone.0111073, PMID: 25350747 PMC4211733

[28] Supabphol S Seubwai W Wongkham S Saengboonmee C. High glucose: an emerging association between diabetes mellitus and cancer progression. J Mol Med. (2021) 99:1175–93. doi: 10.1007/s00109-021-02096-w, PMID: 34036430

[29] Sayed S Faruq O Preya UH Kim JT. Cathepsin S knockdown suppresses endothelial inflammation, angiogenesis, and complement protein activity under hyperglycemic conditions in vitro by inhibiting NF-κB signaling. Int J Mol Sci. (2023) 24:24. doi: 10.3390/ijms24065428, PMID: 36982499 PMC10049538

[30] Adham SA Al Rawahi H Habib S Al Moundhri MS Viloria-Petit A Coomber BL. Modeling of hypo/hyperglycemia and their impact on breast cancer progression related molecules. PLoS One. (2014) 9:e113103. doi: 10.1371/journal.pone.0113103, PMID: 25401697 PMC4234670

[31] Wang H Yan F Cui Y Chen F Wang G Cui W. Association between triglyceride glucose index and risk of cancer: a meta-analysis. Front Endocrinol. (2022) 13:1098492. doi: 10.3389/fendo.2022.1098492, PMID: 36714554 PMC9877418

[32] Song Y Jiang L Han Y Zhang S Li S. Triglyceride-glucose index and glycemic dynamics in pancreatic ductal adenocarcinoma: implications for disease progression and prognosis. J Transl Med. (2024) 22:708. doi: 10.1186/s12967-024-05524-w, PMID: 39080703 PMC11290143

[33] Huo RR Zhai L Liao Q You XM. Changes in the triglyceride glucose-body mass index estimate the risk of stroke in middle-aged and older Chinese adults: a nationwide prospective cohort study. Cardiovasc Diabetol. (2023) 22:254. doi: 10.1186/s12933-023-01983-5, PMID: 37716947 PMC10505325

[34] Wang F He T Wang G Han T Yao Z. Association of triglyceride glucose-body mass index with non-small cell lung cancer risk: a case-control study on Chinese adults. Front Nutr. (2022) 9:1004179. doi: 10.3389/fnut.2022.1004179, PMID: 36313086 PMC9614218

[35] Guo S Zhao Y Jiang Y Ye H Wang Y. Increased pretreatment triglyceride glucose-body mass index associated with poor prognosis in patients with advanced non-small cell lung cancer. Clin Nutr ESPEN. (2024) 59:412–21. doi: 10.1016/j.clnesp.2023.12.018, PMID: 38220404

[36] Dikaiou P Edqvist J Lagergren J Adiels M Björck L Rosengren A. Body mass index and risk of cancer in young women. Sci Rep. (2024) 14:6245. doi: 10.1038/s41598-024-56899-1, PMID: 38485791 PMC10940279

[37] Petrelli F Cortellini A Indini A Tomasello G Ghidini M Nigro O. Association of Obesity with Survival Outcomes in patients with Cancer: a systematic review and Meta-analysis. JAMA Netw Open. (2021) 4:e213520. doi: 10.1001/jamanetworkopen.2021.3520, PMID: 33779745 PMC8008284

[38] Murata D Azuma K Matsuo N Murotani K Matama G Kawahara A. Survival and biomarkers for cachexia in non-small cell lung cancer receiving immune checkpoint inhibitors. Cancer Med. (2023) 12:19471–9. doi: 10.1002/cam4.6549, PMID: 37712645 PMC10587946

